# LC–MS/MS method for simultaneous determination of diethylcarbamazine, albendazole and albendazole metabolites in human plasma: Application to a clinical pharmacokinetic study

**DOI:** 10.1016/j.jpba.2017.12.037

**Published:** 2018-03-20

**Authors:** Yashpal S. Chhonker, Constant Edi, Daryl J. Murry

**Affiliations:** a Dept of Pharmacy Practice, University of Nebraska Medical Center, Omaha, NE 68198, United States; b Fred and Pamela Buffett Cancer Center, University of Nebraska Medical Center, Omaha, NE 68198, United States; c Centre Suisse de Recherches Scientifiques en Côte d’Ivoire (CSRS), 01 BP1303 Abidjan 01, Cote d’Ivoire

**Keywords:** Diethylcarbamazine, Albendazole, Albendazole sulfoxide, LC–MS/MS

## Abstract

Combination therapy with anti-filarial drugs is now widely used for treatment of lymphatic filariasis. A rapid, selective, and sensitive liquid chromatography coupled with tandem mass spectrometry (LC–MS/MS) method was developed and validated for simultaneous quantitation of diethylcarbamazine (DEC), albendazole (ABZ) and albendazole metabolites in human plasma. Separation and detection of analytes were achieved on a reversed phase column (Acquity UPLC^®^BEH C18 column (100 × 2.1 mm, 1.7 μm) with gradient elution using 0.05% formic acid in methanol and 0.05% formic acid as mobile phase. Solid phase extraction was utilized for elution of analytes from the matrix. Thereafter, analytes were monitored by using MS/MS with electrospray ionization source in positive multiple reaction monitoring mode. The MS/MS response was linear over the concentration range from 0.1–200 ng/mL for ABZ and ABZ-ON, 0.5–1000 ng/mL for ABZ-OX and 1–2000 ng/mL for DEC with a correlation coefficient (r^2^) of 0.998 or better. The within- and between-batch precisions (relative standard deviation, % RSD) and the accuracy (% bias) were within the acceptable limits as per FDA guideline. The validated method was successfully applied to the clinical pharmacokinetic study. Due to high sensitivity and low requirement of sample volume, the method will be applicable for therapeutic drug monitoring of this regimen.

## Highlights

• The first LC–MS/MS method of diethylcarbamazine and albendazole along with its active metabolites.
• The method was successfully applied to analyze clinical samples.
• The highly sensitive and selective LC–MS/MS method for routine pharmacokinetic application.
• This method is useful for drug–drug interaction or TDM studies of diethylcarbamazine and albendazole in Lymphatic filariasis therapy.

## 1 Introduction

Lymphatic filariasis (LF), currently infects more than one billion people residing in endemic areas [1]. LF is a mosquito transmitted nematode parasite (*Wuchereria bancrofti*) infection that causes lymphedema, elephantiasis, and hydroceles [2]. LF is treated through mass drug administration (MDA) with diethylcarbamazine (DEC) combined with albendazole (ABZ) and ivermectin (IVM) [3]. The relation between drug exposure, therapeutic response, treatment failure, or side effects related to this triple drug therapy is not currently known. Reported side effects for ABZ range from minor rashes to more severe hepatotoxicity and jaundice [4,5]. Thus, there is a clinical need for therapeutic monitoring of ABZ and DEC along with their metabolites. Moreover, current regimens for lymphatic filiarisis are not completely effective in eradicating microfiliaria. Thus, there is a need for studies to evaluate the relation between drug concentration and effect in this population.

Albendazole is only detectable for a short time in plasma due to its rapid conversion to its major active metabolites such as ABZ-sulfoxide (ABZ-OX) and ABZ-sulfone (ABZ-ON) and subsequently low drug concentrations [6]. Various bioanalytical methods are available for analysis of ABZ and its metabolites in different biological matrices [[7], [8], [9], [10], [11], [12], [13], [14]]. Wojnicz et al. reported a LC–MS/MS method for the quantitation of ABZ and ABZ-OX in 200 μL plasma with a LLOQ of 5 and 10 ng/mL)[10]. Zhang et al. has developed a LC–MS/MS method for ABZ and its metabolites from fish muscle with a LLOQ of 0.1 ng/mL and a required sample weight of 2.0 gm [11]. Xing et al. has a developed a LC–MS/MS method for ABZ and its metabolites with a LLOQ of 0.76–16.67 ng/mL [12]. Rathod et al. developed a LC–MS/MS method for the quantitation of ABZ and ABZ-OX in 100 μL plasma with a LLOQ of 0.3and 3 ng/mL respectively [13]. Pandya et al. has a developed a HPTLC method for ABZ and ABZ-OX, has a LLOQ of and 50 and100 ng/mL[14].

Similarly, bioanalytical methods are available for determination of DEC [[15], [16], [17], [18]], however limitations in these methods still preclude their implementation in PK studies. Lee et al. developed a gas chromatographic method with a LLOQ of 10 ng/mL with a required sample volume of 1 mL of plasma using a NPD detector [18]. Schmidt et al. developed a LC–MS/MS method for the quantitation of DEC in 200 μL plasma (LLOQ; 4 ng/mL) [17].

However, to best of our knowledge, no bioanalytical LC–MS/MS method has been reported for simultaneous analysis of DEC, ABZ and its metabolites (ABZ-OX and ABZ-ON) in human plasma with a LLOQ in the 100 pg/mL range. In order to accurately determine the pharmacokinetics of a drug, sensitive and specific analytical methods are required. To the best of our knowledge, this is the first report on simultaneous determination of DEC, ABZ, ABZ-OX and ABZ-ON using LC–MS/MS in human plasma. Moreover, the present LC–MS/MS assay is highly sensitive (0.1 ng/mL), requires low sample volume (100 μL) and a short analysis time as compared with previously reported assays for each individual drug. The validated bioanalytical method was successfully utilized for the quantitative analysis of DEC, ABZ, ABZ-OX and ABZ-ON, for samples received from clinical pharmacokinetic study of DEC, IVM and ABZ in patients with lymphatic filariasis.

## 2 Materials and methods

### 2.1 Chemicals and materials

**Fig. 1:**
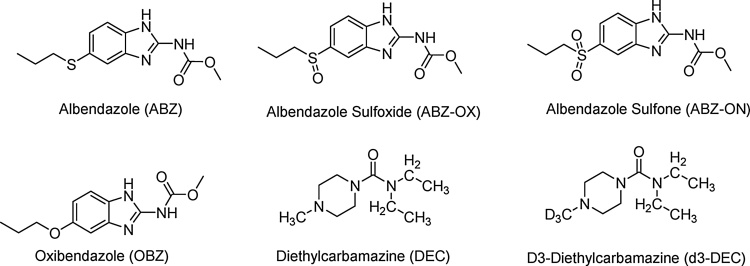
Chemical structures of ABZ, ABZ-OX, ABZ-ON, OBZ, DEC, and D3-DEC.

DEC (purity, ≥98%), ABZ (purity, ≥98%), ABZ-OX (purity, ≥99.9%), ABZ-ON (purity, ≥99.6%), oxibendazole (OBZ) (purity, ≥99.9%), and D3-DEC (purity, ≥98%), (Fig. 1) of pharmaceutical grade were purchased from Sigma-Aldrich, St Louis, MO, USA. HPLC-grade methanol (MeOH), acetonitrile (ACN), formic acid (FA), and acetic acid were obtained from Fisher Scientific (Fair Lawn, NJ). Agilent bond Elute C18, 50 mg per 1 mL cartridges were from Agilent (Santa Clara, CA). Centrifuge tube filters were from Corning Co. (Corning, NY). All other chemical reagents were from Sigma (St. Louis, MO). Ultrapure water was obtained from a water purification system (ThermoFisher Scientific). All other reagents were of analytical grade and obtained from standard commercial suppliers.

### 2.2 Liquid chromatographic and mass spectrometric conditions

A Shimadzu, Nexera UPLC system equipped with two pump (LC‐30 AD) and column oven (CTO‐30AS) along with an auto‐sampler (SIL‐30AC) was used. Mass spectrometric detection was performed on an LC–MS/MS 8060 system (Shimadzu Scientific Instruments, Columbia, MD), equipped with a DUIS source in positive electrospray ionization mode. The MS/MS system was operated at unit resolution in the multiple reaction monitoring (MRM) mode. All chromatographic separations were performed with an Acquity UPLC^®^BEH C18 column (1.7 μm, 100 × 2.1 mm, Part #186002352) equipped with an Acquity UPLC C18 guard column (Waters, Milford, MA).

The mobile phase consisted of 0.05% FA in water (mobile phase A) and 0.05% FA in methanol (MeOH) (mobile phase B), at total flow rate of 0.2 mL/min. The chromatographic separation was achieved using 10 min gradient elution. The initial mobile phase composition was 35% B, increasing to 70% B over 6 min, then increasing to 95% B over 2 min, then held constant at 95% B for 1 min, and finally brought back to initial condition of 35% B in 0.20 min followed by 1-min re-equilibration. The injection volume of all samples was 10 μL.

The compound dependent mass spectrometer parameters, such as temperature, voltage, gas pressure, etc., were optimized by auto method optimization via product ion search for each analyte and the internal standard (IS) using a 1 μg/mL solution in methanol. All analytes were detected in the positive ionization mode utilizing electrospray ionization (ESI) with the following mass spectrometer source settings: nebulizer gas: 2.0 L/min; heating gas: 10 L/min; drying gas: 10 L/min; interface temperature: 375 °C; desolvation line temperature: 250 °C; heat block temperature: 300 °C. The multiple reaction monitoring (MRM) transitions for each analyte and IS, as well as their respective optimum MS parameters, such as voltage potential (Q1, Q3), and collision energy (CE), are shown in Table 1. Data acquisition and quantitation were performed using LabSolutions LCMS Ver.5.6. (Shimadzu Scientific Inc, Columbia MD).

**Table 1:** Summary of MS/MS parameters: precursor ion, fragment ions, voltage potential (Q1), collision energy (CE) and voltage potential (Q3) for analytes.

Analytes	MRM transition *m*/*z* (Q1 → Q3)	Q1 (V)	CE (V)	Q3 (V)	Retention time
ABZ	266.10 > 234.40	−28	−20	−26	6.6
ABZ-OX	282.10 > 240.35	−30	−14	−26	3.8
ABZ-ON	298.10 > 159.30	−15	−35	−17	4.2
DEC	200.15 > 100.45	−20	−15	−11	1.5
OXZ	250.20 > 176.40	−29	−27	−19	4.6
D3-DEC	203.40 > 100.45	−12	−17	−30	1.5

### 2.3 Preparation of stock, calibration standard and quality control sample preparation

The stock solution (1 mg/mL) of DEC, ABZ, ABZ-OX and ABZ-ON were prepared in MeOH. The stock solutions were diluted with methanol to make working standard solutions which were further diluted to prepare the calibration standards (CC) and quality control samples (QCs). Calibration standards were prepared by spiking 10 μL of mixed working standard solution into 100 μL of human plasma to obtain a concentration range of 1–2000 ng/mL for DEC, 0.5–1000 ng/mL for ABZ-OX, 0.1–200 ng/mL for ABZ and ABZ-ON. QC samples at four different concentrations (1, 5, 500 and 1500 ng/mL for DEC; 0.5, 2, 200 and 750 ng/mL for ABZ-OX; 0.1, 0.5, 40 and 150 ng/mL for ABZ and ABZ-ON) viz., lower limit of quantification (LLOQ), low quality control (LQC), middle quality control (MQC) and high quality control (HQC), were prepared separately in five replicates, independent of the calibration standards. A 10 μL spiking of internal standard (IS) from mix internal stock of OBZ and d3-DEC solution in all CC and QCs. All the main stocks, intermediate stocks, spiking calibration, and QCs stock solutions were kept at −20 °C.

### 2.4 Plasma sample preparation

All analytes were extracted from CC, QCs, and human plasma samples by solid phase extraction (SPE) using Agilent bond Elute C18, 50 mg per 1 mL SPE cartridge. Plasma (100 μL) samples were spiked with IS (10 μL), and diluted with water (800 μL), vortexed for 2 min, and then loaded onto SPE cartridges pre-conditioned with MeOH (1 mL), followed by water (1 mL). Loaded cartridges were washed with 5% MeOH (2 mL) and eluted with 1% FA in MeOH (2 mL). For all standards and samples, eluates were evaporated under vacuum at room temperature and reconstituted in 100 μL of 0.1% FA: MEOH (60:40).

### 2.5 Method validation

The developed method was validated according to the guidelines of US Food and Drug Administration (FDA) for industry bioanalytical method validation [19].

#### 2.5.1 Selectivity and specificity

Selectivity was carried out by analyzing the six blank plasma samples spiked with analytes and IS. For specificity, six different lots of blank plasma were evaluated for any interference at the retention times of analytes and IS. They were processed as per extraction procedure.

#### 2.5.2 Sensitivity

The sensitivity of the method was determined from the signal-to-noise ratio (S/N) of the response of analyte in calibration standards. The S/N ratio should be greater than 3 for the limit of detection (LOD) and greater than 10 for the LLOQ. The calibration curves were established by plotting the peak area ratio (analyte/IS) versus concentration for all analytes.

#### 2.5.3 Accuracy and precision

Intra- and inter-day accuracy and precision were evaluated from replicate analyses (*n* = 5) of QC samples containing analytes at different concentrations (LLOQ, LQC, MQC and HQC) prepared on the same day. The precision was calculated in terms of% relative standard deviation (%R.S.D.). The accuracy was expressed as% Bias. The criteria for acceptability of the data included accuracy within ± 15% standard deviation (S.D.) from the nominal values and a precision within ± 15% R.S.D. except for LLOQ, where it should not exceed ± 20% of accuracy as well as precision.

%Bias = (observed concentration −  nominal concentration)/nominal concentration × 100

#### 2.5.4 Recovery and matrix effect

The absolute recovery of all analytes and IS were calculated by comparing the peak area of QC samples (LQC, MQC and HQC, *n* = 5) in plasma with corresponding standard concentrations prepared in reconstitution solvent. The recovery was acceptable if consistent, precise and reproducible [19].

It is complicated to determine matrix effect during bioanalytical method development of multiple compounds in a single run [20,21]. The matrix effect of human plasma constituents over the ionization of all analytes and IS was determined by comparing the responses of the post-extracted plasma standard QC samples (LQC, MQC and HQC, *n* = 5) with the response of analytes from neat standard samples at equivalent concentrations. If the peak area ratio is less than 85% or more than 115%, a matrix effect is implied [22].

#### 2.5.5 Calibration curve

The linearity of the method was evaluated using analyte spiked plasma samples in the concentration range mentioned above using the method of least squares. Five such linearity curves were analyzed. Each calibration curve consisted of a blank sample, a zero sample (blank + IS) and ten non-zero concentrations. The results were fitted to linear regression analysis with the use of 1/*x^2^* weighting factor. The calibration curve had to have a correlation coefficient (r^2^) of 0.998 or better for all analytes. The acceptance criteria for each back calculated standard concentration were ± 15% deviation from the nominal value except at LLOQ which was set at ± 20%.

#### 2.5.6 Carry-over

Carry-over was checked by injecting two zero samples directly after injecting an HQC sample. The response of the first zero sample should be <20% of the response of a processed LLOQ sample.

#### 2.5.7 Dilution integrity

Dilution effect was investigated to ensure that samples could be diluted with blank plasma without affecting the concentration. DEC, ABZ, ABZ-OX and ABZ-ON spiked human plasma samples prepared at 2000 ng/mL concentrations were diluted with pooled human plasma at dilution factors of 2 and 5 in five replicates and analyzed. As part of the validation, five replicates had to comply with both precision of ≤15% and accuracy of 100 ± 15% similar to other QCs samples.

#### 2.5.8 Stability

The auto sampler stability of DEC, ABZ, ABZ-OX and ABZ-ON in plasma was examined at 4 °C for 36 h. The bench-top stability was evaluated at ambient temperature (20 °C) for 8 h using QC samples in three replicates. The storage stability at −80 ± 5 °C over 60 days was also evaluated. The freeze/thaw stability was determined after three freeze/thaw cycles (room temperature to −80 ± 5 °C). All stability studies were performed at LQC, MQC and HQC levels.

### 2.6 Application of the method for clinical drug–drug interaction study samples analysis

Human volunteers (>20 yrs of either sex) presenting with and without *Wuchereria bancrofti* infection in Côte d’Ivoire were included in the study after informed consent was obtained. The clinical study was conducted in accordance with the principles laid down by the World Health Assembly on Ethics in Human Experimentation, the revised version Helsinki Declaration and Good Clinical Practice. Each volunteer was informed of the objectives, nature and possible risks of the trial. All subjects were instructed to fast overnight, and beginning at 07:00 h the next morning, were given by direct observation DEC- citrate 6 mg/kg + ABZ 400 mg + Ivermectin 200 μg/kg with water. Blood samples (2 mL in 3.8% tri-Sodium Citrate 9:1, v/v) were obtained prior to the dose and at 1, 2, 3, 4, 6, 12, 24, 48, 72, 166 h (7 days). The plasma samples were separated by centrifuging the blood samples at 2000 × g for 10 min at 4 °C. All plasma samples were placed into tubes and stored in sealed tubes at −80 ± 5 °C until analysis.

## 3 Results and discussion

### 3.1 Chromatographic and mass spectrometric conditions optimization

To obtain the selectivity and sensitivity for all analytes, several chromatographic and mass spectrometric conditions were optimized. The selection of ionization mode was based on the comparison of obtained sensitivity with ESI and atmospheric pressure chemical ionization (APCI) source. The results showed that ESI operated in positive mode provided increased intensity for the analytes compared to APCI (data not shown). The fragmentation of analytes and IS were auto optimized via precursor ion search of approximately 1000 ng/mL of stock solution for each analyte. The most abundant precursor > product ions in terms of better sensitivity for DEC, ABZ, ABZ-OX and ABZ-ON were found to be *m*/*z* 200.15 > 100.45, 266.10 > 234.40, 282.10 > 240.35, 298.10 > 159.30, respectively. The compound dependent parameters such as voltage potential Q1 (V) and Q3 (V), collision energy (CE), were also optimized to obtain the highest signal intensity for all the analytes and IS (Table 1).

**Fig. 2:**
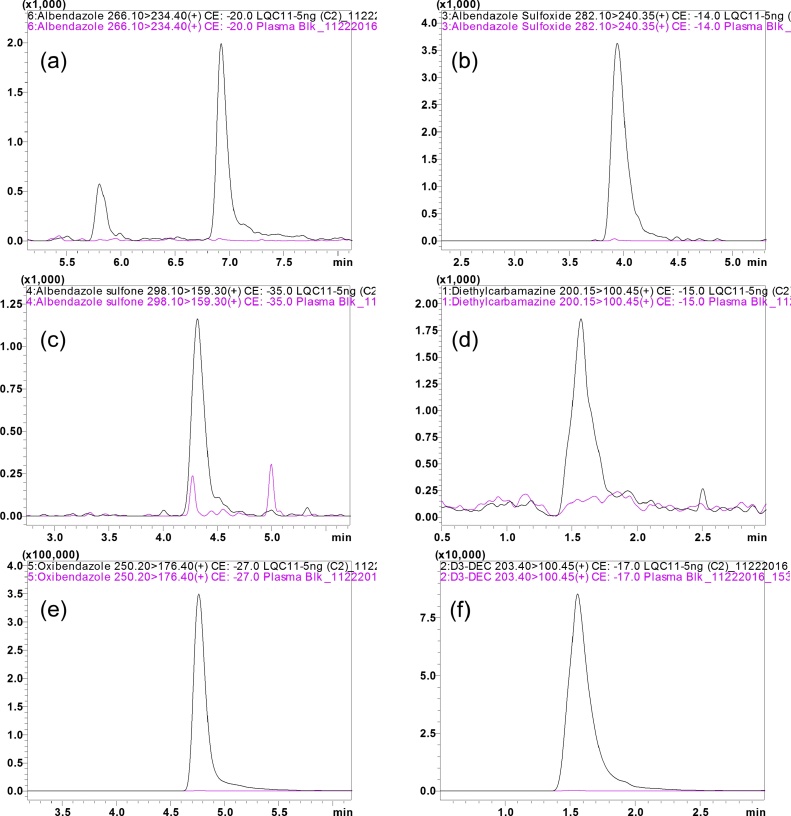
Representative MRM ion- overlay chromatograms (Blank plasma and standard spiked at LQC level) of (a) ABZ, (b) ABZ-OX, (c) ABZ-ON, (d) DEC, (e) OBZ, and (f) D3-DEC.

Chromatographic conditions, including the mobile phase composition and different analytical columns were evaluated and optimized to achieve acceptable resolution and symmetrical peak shapes of the analytes, as well as a short run time. Different solvent systems such as ACN and MeOH with various buffers like ammonium acetate and ammonium formate with different pH range, composition and different flow rates were tried. The suitability and robustness of the method was evaluated by using different varieties of reverse phase HPLC columns ranging from 50 mm to 150 mm in length (data not shown). Complete and rapid chromatographic resolution of analytes and IS was achieved on Acquity UPLC^®^ BEH C18 column (1.7 μm, 100 × 2.1 mm Waters, Milford MD) equipped with an Acquity UPLC C18 guard column (Waters, Milford MD). Symmetrical peak shape was obtained using 0.05% FA in MeOH: 0.05% FA at a flow rate of 0.2 mL/min. with 40 °C as the column temperature. The representative overlay chromatograms with blank plasma show no interference of endogenous compounds at the retention time of ABZ, ABZ-OX and ABZ-ON, for samples spiked at LQC concentration Fig. 2. Further, the reproducibility (%CV) in the measurement of retention time for the analytes was 0.5% for 100 injections. Post column infusion further substantiated the absence of matrix effects with no signal enhancement or suppression at the retention time of ABZ, ABZ-OX, ABZ-ON, OBZ, DEC and D3-DEC.

OBZ was selected as the IS for ABZ, ABZ-OX, ABZ-ON, and D3-DEC for DEC. Both compounds had similar chromatographic behavior without prolonging the analysis time and similar ionization response in ESI mass spectrometry to that of analytes. In addition, the extraction recovery of OBZ (84.48%) and D3-DEC (42.56%) were satisfactory and stable during the whole analytical process.

### 3.2 Assay validation

#### 3.2.1 Specificity and selectivity

The specificity of the intended method was established by screening the blank (drug free) plasma from six independent sources. Chromatograms of six batches blank plasma contained no co-eluting peaks that were >20% of analytes area at LLOQ level and no co-eluting peaks >5% of the area of IS. The retention time for DEC, ABZ, ABZ-OX, ABZ-ON, OBZ and D3-DEC were 1.49, 1.39 and 2.18 min, respectively. The representative UPLC overlay chromatogram with blank plasma, samples spiked at LQC concentration was shown in Fig. 2 indicating that no endogenous interfering peaks were observed at the retention times of analytes and IS. Analytes and IS peak showed less variability with a relative standard deviation (R.S.D.) well within the acceptable limit of ± 5%.

#### 3.2.2 Accuracy and precision

Intra- and inter-day accuracy and precision values for the QC samples are summarized in Table 2. The results showed that the bioanalytical method is accurate, as the bias ranged from −14.8 to (−)13.0%, was within the acceptance limits of ± 20% of the theoretical value at LLOQ and ± 15% at all other concentration levels. The intra- and inter-day precision (% RSD) values were ranged from 3.5 to 13.9%, indicating good assay precision. These data confirm that the method described has a satisfactory accuracy and precision for the quantitation of all the analytes.

**Table 2:** Assessment of accuracy (% Bias) and precision (% R.S.D.) of DEC, ABZ, ABZ-OX and ABZ-ON in human plasma.

Drug	Precision (% RSD)								Accuracy (% Bias)							
	LLOQ		LQC		MQC		HQC		LLOQ		LQC		MQC		HQC	
	Inter-day	Intra-day	Inter-day	Intra-day	Inter-day	Intra-day	Inter-day	Intra-day	Inter-day	Intra-day	Inter-day	Intra-day	Inter-day	Intra-day	Inter-day	Intra-day
ABZ	8.5	3.6	7.1	5.5	6.3	5.6	7.9	4.5	14.8	10.7	9.3	−0.2	−0.6	−0.9	−3.2	−12.3
ABZ-OX	11.0	8.5	3.5	5.7	7.3	7.8	6.1	4.3	−13.0	0.9	6.4	14.1	5.3	11.6	3.6	−3.6
ABZ-ON	8.9	11.0	6.1	8.1	4.6	4.1	5.1	4.9	−7.8	1.0	−7.7	−5.1	−0.3	8.4	−9.3	−6.9
DEC	13.9	10.7	8.2	8.8	5.7	4.4	5.7	5.2	−3.9	3.9	2.5	−0.6	−0.2	−0.9	2.1	0.8

#### 3.2.3 Recovery and matrix effect

The recovery of analytes was calculated from the spiked plasma samples at LQC, MQC and HQC concentrations. The absolute mean recoveries for the average of all three control samples were 71.27 ± 3.13, 100.05 ± 2.59, 113.24 ± 3.79 and 66.23 ± 2.77% for ABZ, ABZ-OX, ABZ-ON and DEC, respectively, across the concentrations (Table 3). The% coefficient of variation (CV) was ± 15% among the mean recoveries at LQC, MQC and HQC levels, proved to be consistent, precise, and reproducible. In addition, the extraction recovery of ISs were 84.48% and 42.56% for OBZ and D3-DEC, respectively. In spite of relatively low recoveries D3-DEC (∼42%), measurement of these analytes proved to be consistent, precise, and reproducible.

**Table 3:** Mean extraction recoveries of the DEC, ABZ, ABZ-OX and ABZ-ON at LQC, MQC and HQC level in plasma from human plasma.

Analyte	% Extraction recoveries								
	LQC			MQC			HQC		
	Mean	SD	%CV	Mean	SD	%CV	Mean	SD	%CV
ABZ	67.77	5.47	8.07	75.31	2.94	3.90	71.49	8.83	12.35
ABZ-OX	103.11	5.98	5.80	97.34	10.31	10.59	101.05	7.96	7.88
ABZ-ON	116.54	6.05	5.19	108.67	14.2	13.07	115.24	5.54	4.81
DEC	67.49	3.99	5.91	62.55	3.98	6.36	69.17	3.08	4.45

The matrix effects calculated were in the range of 92.12–102.20%. Therefore, ion suppression or enhancement from human plasma was negligible under the current conditions.

#### 3.2.4 Calibration curve and carry-over

Calibration curve was linear over the concentrations range 0.1-200 for ABZ and ABZ-ON, 0.5–1000 ng/mL for ABZ-OX and 1–2000 ng/mL for DEC. The average determination coefficient was >0.998 or better. The lowest concentration with R.S.D.<20% was taken as LLOQ. The different dynamic ranges were used because the different analytes had different sensitivity, plasma concentrations in study samples, and/or signal linearity. For example, the LLOQ of DEC was 1 ng/mL in plasma, not due to limitations in detection sensitivity (limit of detection 0.1 ng/mL), but rather due to the relatively high plasma concentrations in study samples. The analytes showed no significant peak (<20% of the LLOQ) in zero samples injected after the HQC samples, thus there was no significant carry over effect.

#### 3.2.5 Dilution integrity

The precision for dilution integrity of 1:2 and 1:5 dilutions were within acceptable limit for all analytes, which is within the acceptance limits of ± 15% for precision (CV) and 85.0–115.0% for accuracy. The results suggested that plasma samples whose concentrations above upper limit of quantitation can be determined by appropriate dilution.

#### 3.2.6 Stability

Stability was demonstrated under a wide variety of conditions and the results of the stability studies were enumerated in Table 4. In the different stability experiments carried out, viz., long-term storage, bench-top, autosampler, and freeze/thaw stability, the mean percentage nominal values of the analytes were found to be within ± 15% of the predicted concentrations for the analytes at their LQC, MQC and HQC levels. Thus, the results were found to be within the acceptable limits during the entire validation.

**Table 4:** Average Mean stability recoveries of the DEC, ABZ, ABZ-OX and ABZ-ON at different storage conditions.

Analyte	% Stability recoveries (Mean ± SD)			
	Freeze-thaw (−80 ± 5 °C after three cycle)	Long-term (−80 ± 5 °C, 60 days)	Auto-sampler (4 °C, 36 h)	Bench-top (20 °C, 8 h)
ABZ	93.84 ± 5.25	101.18 ± 10.18	106.88 ± 7.60	90.30 ± 6.60
ABZ-OX	104.98 ± 6.23	102.68 ± 11.82	106.42 ± 6.62	108.25 ± 8.66
ABZ-ON	110.26 ± 5.10	103.87 ± 10.15	99.27 ± 6.33	109.45 ± 5.10
DEC	112.9 ± 2.15	103.35 ± 10.77	111.27 ± 4.22	113.78 ± 1.87

### 3.3 Application of the method for clinical samples analysis

**Fig. 3:**
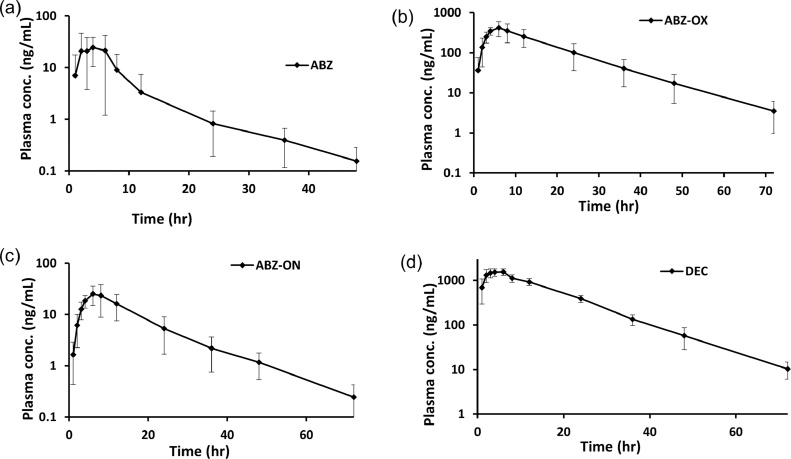
Plasma Concentration-Time profile of (a) ABZ, (b) ABZ-OX, (c) ABZ-ON, (d) DEC in patients receiving the ABZ, and DEC (Mean ± SD, n#7).

The method was successfully applied to support an open-label cohort study of treatment naïve Wuchereria bancrofti-infected and non-infected adults residing in Agboville district of Côte d’Ivoire. The study protocol and related documents were approved by institutional review boards in Cleveland, USA (University Hospitals Cleveland Medical Center IRB #03-16-09) and in Côte d’Ivoire (Comité National d’Ethique et de la Recherche, CNER, N/Ref:022/MSLS/CNER-kp). This trial is registered at Clinicaltrials.gov (NCT02845713). Briefly, healthy-volunteers and patients with active lymphatic filiarsis infection received a three-drug regimen containing DEC, ABZ and IVM. Serial blood samples were obtained and analyzed using this newly developed assay for DEC and ABZ in order to evaluate the effect of disease state on the pharmacokinetics of this drug regimen. Data from seven subjects was selected to include for the pharmacokinetic data. The Fig. 3 shows a representative plasma concentration time profile of the DEC, ABZ such ABZ-OX and ABZ-ON (Mean ± SD, n#7). Both metabolites of ABZ, ABZ-OX and ABZ-ON were detected in therapy up to 72 h in human plasma after single dose.

## 4 Conclusion

The developed LC–MS/MS method is useful in therapeutic drug monitoring of ABZ and DEC, which is the widely preferred Lymphatic filariasis therapy. The method is suitable for high-throughput bioanalysis of DEC, ABZ, ABZ-OX and ABZ-ON owing to a run time of 8 min per sample. Assay performance, including linearity, precision and sensitivity were satisfactory for routine pharmacokinetic application. The method is readily applicable for therapeutic drug monitoring of DEC and ABZ in routine clinical use. The improved level of quantitation allowed us to monitor drug concentrations for 48 h for all studied compounds. Moreover, the improved sensitivity will allow for the extension of these studies in neonates and children where blood volume for bioanalytical samples may be limited. This method may also enable the identification of patients at increased risk of ABZ toxicities by characterizing the concentrationof the metabolites present in plasma. Therefore, the current LC–MS/MS method provides a valuable tool to improve the efficacy and safety of DEC and ABZ therapy.

## Conflict of interest

There is no conflict of interest to disclose.

## References

[1] Taylor M.J., Hoerauf A., Bockarie M. (2010). Lymphatic filariasis and onchocerciasis. Lancet.

[2] Nutman T.B. (2013). Insights into the pathogenesis of disease in human lymphatic filariasis. Lymphat. Res. Biol..

[3] Hussain M.A., Sitha A.K., Swain S., Kadam S., Pati S. (2014). Mass drug administration for lymphatic filariasis elimination in a coastal state of India: a study on barriers to coverage and compliance. Infect. Dis. Poverty.

[4] Marin Zuluaga J.I., Marin Castro A.E., Perez Cadavid J.C., Restrepo Gutierrez J.C. (2013). Albendazole-induced granulomatous hepatitis: a case report. J. Med. Case Rep..

[5] Sivgin S., Eser B., Kaynar L., Kurnaz F., Sivgin H., Yazar S., Cetin M., Unal A. (2013). Encephalitozoon intestinalis: a rare cause of diarrhea in an allogeneic hematopoietic stem cell transplantation (HSCT) recipient complicated by albendazole-related hepatotoxicity. Turk. J. Haematol..

[6] Edwards G., Breckenridge A.M. (1988). Clinical pharmacokinetics of anthelmintic drugs. Clin. Pharmacokinet..

[7] Wojnicz A., Cabaleiro-Ocampo T., Román-Martínez M., Ochoa-Mazarro D., Abad-Santos F., Ruiz-Nuño A. (2013). A simple assay for the simultaneous determination of human plasma albendazole and albendazole sulfoxide levels by high performance liquid chromatography in tandem mass spectrometry with solid-phase extraction. Clin. Chim. Acta.

[8] Grabowski T., Jaroszewski J.J., Swierczewska A., Sawicka R., Maslanka T., Markiewicz W., Ziolkowski H. (2011). Application of ultra-performance columns in high-performance liquid chromatography for determination of albendazole and its metabolites in turkeys. Biomed. Chromatogr.: BMC.

[9] Hou X.L., Chen G., Zhu L., Yang T., Zhao J., Wang L., Wu Y.L. (2014). Development and validation of an ultra high performance liquid chromatography tandem mass spectrometry method for simultaneous determination of sulfonamides, quinolones and benzimidazoles in bovine milk. J. Chromatogr. B: Analyt. Technol. Biomed. Life Sci..

[10] Wojnicz A., Cabaleiro-Ocampo T., Román-Martínez M., Ochoa-Mazarro D., Abad-Santos F., Ruiz-Nuño A. (2013). A simple assay for the simultaneous determination of human plasma albendazole and albendazole sulfoxide levels by high performance liquid chromatography in tandem mass spectrometry with solid-phase extraction. Clin. Chim. Acta.

[11] Zhang X., Xu H., Zhang H., Guo Y., Dai Z., Chen X. (2011). Simultaneous determination of albendazole and its metabolites in fish muscle tissue by stable isotope dilution ultra-performance liquid chromatography tandem mass spectrometry. Anal. Bioanal. Chem..

[12] Li L., Xing D.-X., Li Q.-R., Xiao Y., Ye M.-Q., Yang Q. (2014). Determination of albendazole and metabolites in silkworm bombyx mori hemolymph by ultrafast liquid chromatography tandem triple quadrupole mass spectrometry. PLoS One.

[13] Rathod K.R., Patel H.N., Mistri A.G., Jangid P.S., Shrivastav M. (2016). Liquid chromatography–tandem mass spectrometry method for simultaneous determination of albendazole and albendazole sulfoxide in human plasma for bioequivalence studies. J. Pharm. Anal..

[14] Pandya J.J., Sanyal M., Shrivastav P.S. (2017). Simultaneous densitometric determination of anthelmintic drug albendazole and its metabolite albendazole sulfoxide by HPTLC in human plasma and pharmaceutical formulations. Biomed. Chromatogr..

[15] Zhang D., Park J.A., Kim D.S., Kim S.K., Shin S.J., Shim J.H., Shin S.C., Kim J.S., Abd El-Aty A.M., Shin H.C. (2016). A simple extraction method for the simultaneous detection of tetramisole and diethylcarbamazine in milk, eggs, and porcine muscle using gradient liquid chromatography-tandem mass spectrometry. Food Chem..

[16] Bolla S., Boinpally R.R., Poondru S., Devaraj R., Jasti B.R. (2002). Pharmacokinetics of diethylcarbamazine after single oral dose at two different times of day in human subjects. J. Clin. Pharmacol..

[17] Schmidt C.L., King E.K., Thomsen P.M., Siba N., Sanuku L. (2014). Liquid chromatography?mass spectrometry analysis of diethylcarbamazine in human plasma for clinical pharmacokinetic studies. J. Pharm. Biomed. Anal..

[18] Lee S., Casteel D.A., Fleckenstein L. (1997). Specific gas chromatographic analysis of diethylcarbamazine in human plasma using solid-phase extraction. J. Chromatogr. B: Biomed. Sci. Appl..

[19] (2013). U. Food, Drug Administration Centre for Drug Evaluation and Research (FDA). Guidance for Industry-Bioanalytical Method Validation.

[20] Van Eeckhaut K., Lanckmans S., Sarre I., Smolders Y. (2009). Validation of bioanalytical LC–MS/MS assays: evaluation of matrix effects. J. Chromatogr. B.

[21] Taylor P.J. (2005). Matrix effects: the Achilles heel of quantitative high-performance liquid chromatography–electrospray–tandem mass spectrometry. Clin. Biochem..

[22] Matuszewski M.L.C.B.K., Chavez-Eng C.M. (1998). Matrix effect in quantitative LC–MS/MS analyses of biological fluids: a method for determination of finasteride in human plasma at picogram per milliliter concentrations. Anal. Chem..

